# Breast Cancer Classification Based on Tumor Budding and Stem Cell-Related Signatures Facilitate Prognosis Evaluation

**DOI:** 10.3389/fonc.2021.818869

**Published:** 2022-01-10

**Authors:** Zhenxian Xiang, Qiuming He, Li Huang, Bin Xiong, Qingming Xiang

**Affiliations:** ^1^ Department of Gastrointestinal Surgery, Zhongnan Hospital of Wuhan University, Wuhan, China; ^2^ Department of Gastric and Colorectal Surgical Oncology, Zhongnan Hospital of Wuhan University, Wuhan, China; ^3^ Hubei Key Laboratory of Tumor Biological Behaviors, Wuhan, China; ^4^ Hubei Cancer Clinical Study Center, Wuhan, China; ^5^ Department of Pathology, Tongji Hospital, Tongji Medical College, Huazhong University of Science and Technology, Wuhan, China; ^6^ Department of Radiation and Medical Oncology, Zhongnan Hospital of Wuhan University, Hubei Key Laboratory of Tumor Biological Behaviors & Hubei Cancer Clinical Study Center, Wuhan, China

**Keywords:** CSCs, ALDH1A1, CD24, CD44, breast cancer, tumor budding, prognosis, EMT

## Abstract

**Background:**

Tumor budding (TB) is emerging as a prognostic factor in multiple cancers. Likewise, the stemness of cancer cells also plays a vital role in cancer progression. However, nearly no research has focused on the interaction of TB and tumor stemness in cancer.

**Methods:**

Tissue microarrays including 229 cases of invasive breast cancer (BC) were established and subjected to pan-cytokeratin immunohistochemical staining to evaluate molecular expression. Univariate and multivariate analyses were applied to identify prognostic factors of BC, and the Chi-square test was used for comparison of categorical variables.

**Results:**

High-grade TB was significantly associated with T stage, lymph node metastasis, tumor node metastasis (TNM) stage, epithelial-mesenchymal transition, and poor disease-free survival (DFS) of BC patients. We also found that the prognostic value of TB varied widely among different subtypes and subgroups. Cox regression analysis then showed that TB grade was an independent prognostic factor. Moreover, cancer stem cell (CSC) markers CD44 and ALDH1A1 were significantly higher in high-grade TB tumors. Consequently, patients were classified into high CSC score subgroup and low CSC score subgroups. Further research found that CSC scores correlated with clinicopathological features and DFS of BC patients. Based on TB grade and CSC scores, we classified BC patients into TB_low_-CSCs_low_ (type I), TB_low_-CSCs_high_ (type II), TB_high_-CSCs_low_ (type III), and TB_high_-CSCs_high_ (type IV) subgroups. Survival analysis showed that patients in the type I subgroup had the best DFS, whereas those in the type IV subgroup had the worst DFS. Finally, a TB-CSC-based nomogram for use in BC was established. The nomogram was well calibrated to predict the probability of 5-year DFS, and the C-index was 0.837. Finally, the area under the curve value for the nomogram (0.892) was higher than that of the TNM staging system (0.713).

**Conclusion:**

The combination of TB grade with CSC score improves the prognostic evaluation of BC patients. A novel nomogram containing TB grade and CSC score provides doctors with a candidate tool to guide the individualized treatment of cancer patients.

## Introduction

Breast cancer (BC), which has the highest incidence of any female cancer worldwide, is one of the significant risk factors affecting women’s health (1). Owing to cancer heterogeneity and individual differences, BC patients show variation in prognosis. That is to say, despite a favorable overall survival rate, the recurrence rate of BC within 15 years exceeds 40% (2). Therefore, individualized cancer therapy appears to be important to maximize therapeutic effects and improve quality of life. Standardized and reproducible biomarkers, which could be applied to predict tumor progression, are a cornerstone of individualized cancer therapy.

Tumor budding (TB), first introduced in colorectal cancer and typically defined as the formation of single malignant cells or cell clusters of fewer than five malignant cells at the invasive tumor front (3), is an emerging prognostic biomarker in solid cancers (4, 5). The 2019 World Health Organization (WHO) classification of colorectal cancer introduce TB as a second major grading criterion (6). Additionally, the prognostic value of TB in CRC is emphasized by the inclusion of this feature as an additional prognostic factor for this disease in the tumor-node-metastasis (TNM) classification of 2017 and WHO classification of 2019 (3, 6, 7). Besides, TB is also a novel prognostic indicator independent of tumor stage and grade in esophageal, gastric (8), bladder (9), and pancreatic tumors (10, 11). Owing to the lack of standardized scoring systems and large-scale studies, whether TB represents an additional prognostic factor in BC requires further research.

The concept of cancer stem cells (CSCs) was first formulated in 1800 (12) and refers to a unique subset of cells with elevated self-renewal, differentiation, and proliferation abilities (13). Because of their “stem-like” properties commonly shared with normal tissue stem cells, these cells are termed CSCs. In acute myeloid leukemia, researchers first found the clear evidence of CSCs being an essential tumor-initiating subset of cancer cells (14, 15). Since then, similar tumor-initiating subpopulations have been identified in various types of cancers *via* different CSC cell surface markers or side population (SP) analysis (16–18). Accumulating evidence demonstrates that breast CSCs originate from either normal mammary stem cells or mammary epithelial cells by epithelial-mesenchymal transition (EMT) (19). In addition, CSCs have been shown to maintain the dormant state of BC during chemotherapy and confer resistance to anoikis, causing BC recurrence, metastasis, and therapy resistance (20, 21). Numerous CSC surface markers (CD44, CD24, and ALDH1A1) (22) that can be used to assess prognosis have been identified in BC (23). As is known to all, TB is a complex biological phenomenon that is closely related to increased tumor cell dissociation, migration, and infiltration. EMT, which is the first step of TB (24, 25), has been shown to play a prominent role in tumor cell dissociation. Subsequently, some detached cancer cells could acquire stem cell phenotype to adapt to a hypoxic environment (26, 27). Thus, TB cells may acquire CSC phenotype to realize distant metastasis and colonization (28). However, whether a combination of TB and CSC markers could be used to estimate the outcomes of BC more precisely remains to be explored.

This study found that high-grade TB was correlated with the TNM stage, lymph node metastasis (LNM), and EMT of BC. Furthermore, we identified TB as an independent prognostic factor and showed that high-grade TB was correlated with worse disease-free survival (DFS) of cancer patients. Subsequently, we verified that CSC scores were correlated with tumor progression and TB. A novel nomogram based on TB and CSC score was constructed and shown to improve the prognostic evaluation of BC. The defined subtype may provide guidance for individualized treatment of cancer patients.

## Materials and Methods

### Patients and Tissue Arrays

Tumor tissue microarrays (TMAs), containing 240 cases of formalin-fixed paraffin-embedded invasive BC tissues from Hubei Cancer Hospital, were constructed (January 2002–December 2006). Eleven cases of tumor tissues were excluded due to substandard quality or incomplete information. Finally, 229 cases of specimens were enrolled in our research. Major pathological parameters, including tumor size, location, LNM, estrogen receptor (ER) status, progesterone receptor (PR) status, human epidermal growth factor receptor 2 (HER2) status, neoadjuvant therapy, and postoperative treatment, were collected from the medical record. The Research Ethics Committee of Wuhan University (Wuhan, Hubei, China) approved this study. Informed consent was obtained from all participating patients.

### Immunohistochemistry

Slides were baked in a 65°C oven for 2 h. Slides were then deparaffinized by xylene. After rehydration, we used the citrate buffer to retrieve the antigen. Being incubated with 3% hydrogen peroxide (Merck, Darmstadt, Germany) for 20 min, slides were blocked with 0.5% BSA (Beyotime, China) for 20 mins at 37°C. Next, sections were incubated overnight with primary antibody rabbit anti-pankeratin, anti-CD44 (1:100, CST 37259S), anti-ALDH1A1 (1:400, CST 36671S), and anti-CD24 (1:300, CST 9705S), anti-E-cadherin (1:400, CST 3195S, China), and anti-vimentin (1:300, CST 5741S, China). The next day, sections were incubated with secondary antibody labeled with horseradish peroxidase (HPR) for 30 min. Finally, slides were stained with diaminobenzidine and hematoxylin.

### TB Assessment and IHC Score

According to the International TB Consensus Conference (ITBCC) 2016 (29), standard criteria for TB assessment was made in colorectal cancer. Pan-cytokeratin immuno-histochemistry (IHC), which could highlight tumor buds and improve the interobserver agreement, was chosen to assess TB (3). In brief, TB is assessed in one 0.785 mm^2^ hotspot at the invasive front. The TB was evaluated and scored by pathologist (Qingming Xiang and Li Huang). CD44, ALDH1A1, CD24, E-cadherin, and vimentin expressions were calculated as the product of percentage expressing cells (calculated by counting the number of positive tumor cells among at least 1,000 tumor cells for each tissue section manually) multiplied by mean intensity (0 to 2+). All IHC results were independently scored by two pathologists (Qingming Xiang and Li Huang). The X-tile software was used to select the best cutoff value for E-cadherin expression, vimentin expression, and TB numbers.

### Statistical Analysis

IBM SPSS 24.0 (Chicago, IL, USA) was used to perform statistical analyses. Univariate and multivariate analyses were used to identify prognostic factors, and the Chi-square test was used to calculate significant differences between categorical variables. R 3.6.3 software (https://cran.r-project.org/) was used to construct heatmap and the nomogram (“DynNom” package). *p*-values less than 0.05 were considered statistically significant.

## Result

### Patient Characteristics and Pathological Examination

**Figure 1:**
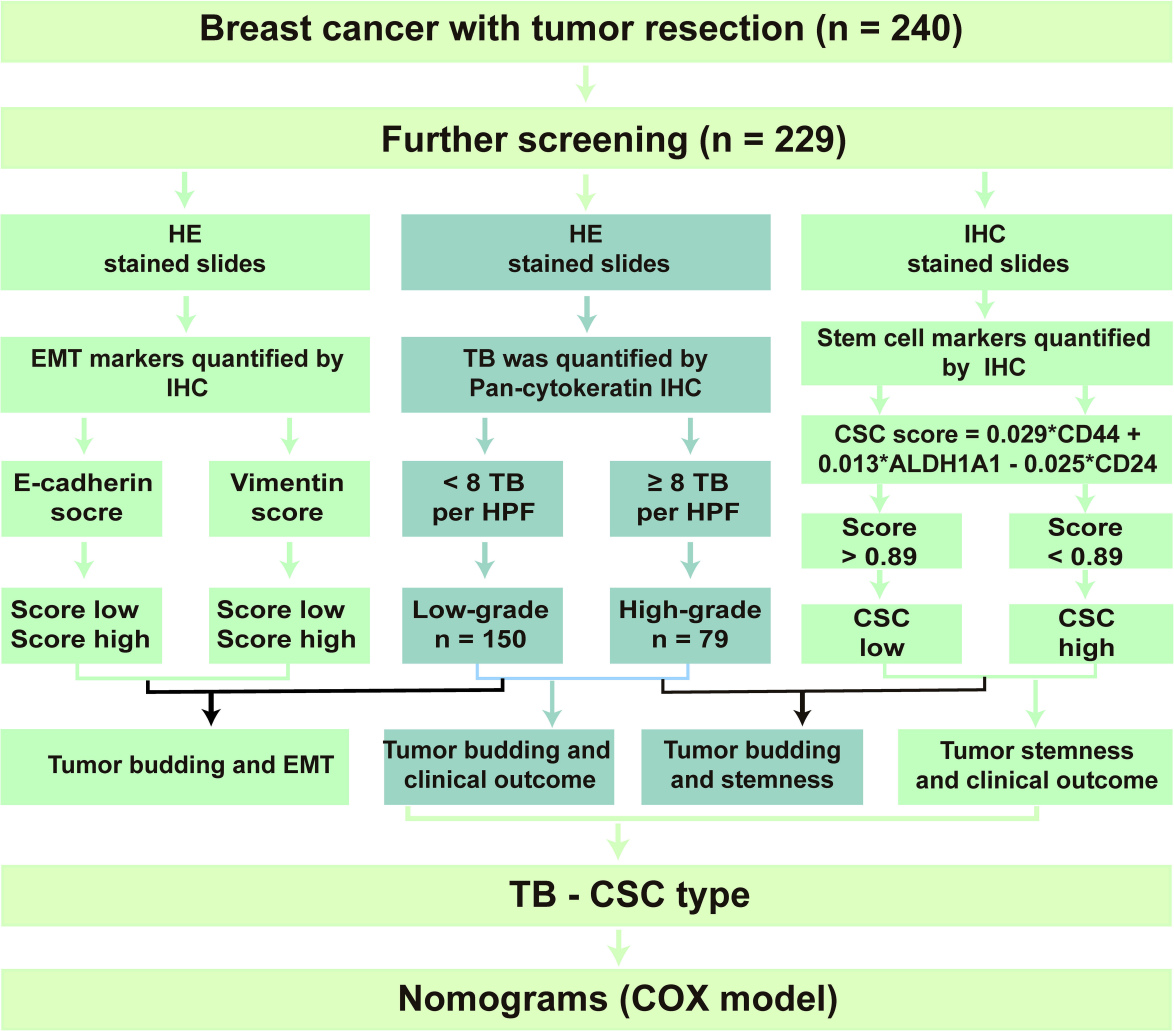
Flow chart of the study design.

After screening, 229 patients with invasive BC were enrolled in the present research. The clinicopathological features of these 229 patients are shown in **Table 1**. The details of the study design and a flow chart are shown in **Figure 1**. We divided the participants into two groups: those that had tumor recurrence (86 patients) and those that did not have tumor recurrence. In addition, 62% of participants were under 50 and 100 (44%) had gone through menopause. Neoadjuvant therapy had been conducted in 44% of participants, and 190 patients had undergone postoperative chemoradiotherapy.

**Table 1:** Basal characteristics of 229 patients with invasive BC.

Characteristics	Total cohort	Without recurrence	With recurrence
		*N* (%)	*N* (%)
**Total cases**	229	143 (100%)	86 (100%)
**Age (years)**			
≤50	142 (62%)	89 (62%)	53 (62%)
>50	87 (38%)	54 (38%)	33 (38%)
**Menopausal status**			
Premenopausal	129 (56%)	86 (60%)	43 (50%)
Postmenopausal	100 (44%)	57 (40%)	43 (50%)
**T stage**			
T1	30 (13%)	27 (19%)	3 (3%)
T2	156 (68%)	100 (70%)	56 (65%)
T3	43 (19%)	16 (11%)	27 (31%)
**LNM**			
N (−)	102 (45%)	86 (60%)	16 (19%)
N (+)	127 (55%)	57 (40%)	70 (81%)
**Tumor differentiation**			
Well	37 (16%)	34 (24%)	3 (3%)
Moderate	134 (59%)	97 (68%)	37 (43%)
Poor	58 (25%)	12 (8%)	46 (53%)
**ER**			
Negative	127 (55%)	65 (45%)	62 (72%)
Positive	102 (45%)	78 (55%)	24 (28%)
**PR**			
Negative	127 (55%)	70 (49%)	57 (66%)
Positive	102 (45%)	73 (51%)	29 (34%)
**TNM stage**			
I	14 (6%)	14 (10%)	0 (0%)
II	147 (64%)	107 (75%)	40 (47%)
III	68 (30%)	22 (15%)	46 (53%)
**HER2 status**			
Negative	171 (75%)	115 (80%)	56 (65%)
Positive	58 (25%)	28 (20%)	30 (35%)
**Neoadjuvant therapy**			
CMT	101 (44%)	51 (36%)	50 (58%)
No treatment	28 (56%)	92 (64%)	36 (42%)
**Postoperative treatment**			
CMT	141 (62%)	97 (68%)	44 (51%)
CMT+R	49 (21%)	20 (14%)	29 (34%)
No treatment	39 (17%)	26 (18%)	13 (15%)
**TB**			
Low-grade TB	150	109	41
High-grade TB	79	34	45

LNM, lymph node metastasis; ER, estrogen receptor; PR, progesterone receptor; TNM, tumor node metastasis; HER2, human epidermal growth factor receptor 2; CMT, chemotherapy; CMT+R, chemotherapy + radiotherapy.

ER-positive and PR-positive patients were found in 102 (55%) and 102 (55%) cancer patients, respectively. In addition, there were 58 (25%) cases of HER2-positive cancer patients. Of the 229 tumors, 192 were classified as showing moderate differentiation or poor differentiation. A total of 127 tumors were LNM positive, and the T stage of most tumors (68%) was T2. TNM stage was classified according to the American Joint Committee on Cancer guidelines, and 64% of patients were classified as stage II. Finally, 79 cases of tumors were identified as high-grade TB, and 150 cases were identified as low-grade TB.

### Budding Quantification and Its Relationship With Patients’ Clinical Outcome

**Figure 2:**
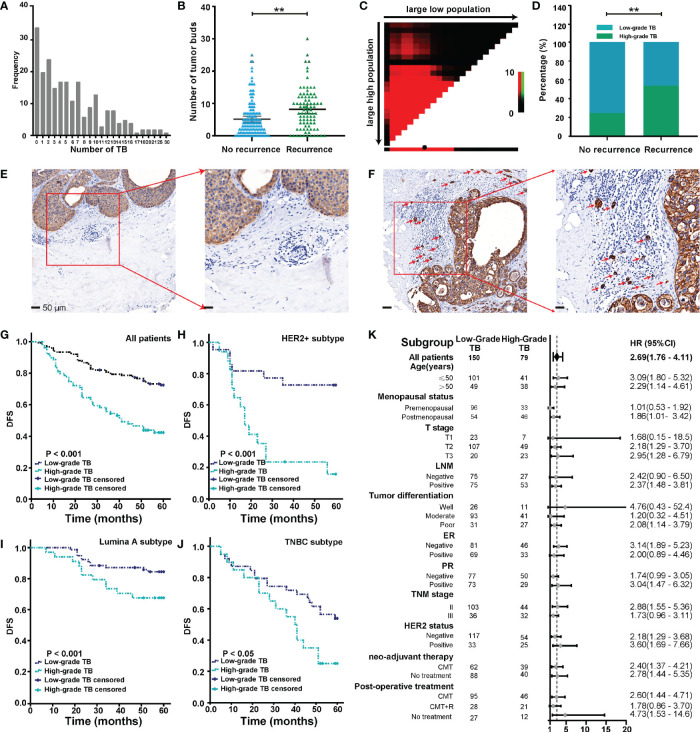
Budding quantification and its relationship with clinical outcome. **(A)** Distribution of tumor buds in 229 cases of BC. **(B)** TB score in recurrent and nonrecurrent groups. **(C)** Analyses to define the optimal cutoff value for TB. **(D)** TB grade in recurrent and nonrecurrent groups. **(E, F)** Representative images of low-grade TB **(E)** and high-grade TB **(F)**. Red arrows point to tumor buds. **(G)** The Kaplan-Meier survival curve shows the DFS of BC after stratification by TB grade. **(H–J)** The Kaplan-Meier survival curve shows DFS of different BC subtypes after stratification by TB. **(K)** The forest map shows the prognostic significance of TB in different subgroups. ^**^ *p* < 0.01.

As shown in **Figure 2A**, we observed a wide variability of TB numbers in BC, ranging from 0 to 30. The median value and mean value of TB numbers in the recurrence cohort were 7 and 8.2, respectively. Also, the median value and mean value of TB numbers in the no-recurrence cohort were 4 and 5.2, respectively. In addition, we found that the number of TB was larger in the recurrence group than the no-recurrence group, and the difference between the two groups was statistically significant (**Figure 2B**).

All possible cutoff values obtained from X-Tile (version 3.6.1) were examined with respect to their ability to predict tumor progression (30), and a budding count of eight was defined as the optimal cutoff value (**Figure 2C**). As shown in **Figure 2D**, we found that the rate of high-grade TB in the recurrence group was higher than the no-recurrence group. This result indicated that tumors with high-grade TB were more likely to recur. Representative pan-cytokeratin IHC images of low-grade TB and high-grade TB are shown in **Figures 2E, F**, respectively. TB was significantly associated with age and menopausal status (**Table 2**). Importantly, the rate of high-grade TB was significantly higher in tumors with higher T stage, LNM positivity, and advanced TNM stage (**Table 2**). However, no significant association was found between TB (high- or low-grade) and tumor differentiation, ER expression, PR expression, HER2 status, neoadjuvant therapy, or postoperative treatment (**Table 2**). These results demonstrate that TB might involve in cancer progression.

**Table 2:** The relationship between TB, CSC score, and major clinicopathological characteristics of BC patients.

Characteristics	Low-grade TB	High-grade TB	*p*-value	Low CSC score	High CSC score	*p*-value
	*N* (%)	*N* (%)		*N* (%)	*N* (%)	
**Total cases**	150	79		114 (100%)	115 (100%)	
**Age (years)**						
≤50	101 (67%)	41 (52%)		75 (66%)	67 (58%)	
>50	49 (33%)	38 (48%)	***0.022***	39 (34%)	48 (42%)	0.241
**Menopausal status**						
Premenopausal	96 (64%)	33 (42%)		71 (62%)	58 (50%)	
Postmenopausal	54 (36%)	46 (58%)	***0.001***	43 (38%)	57 (50%)	0.071
**T stage**						
T1	23 (15%)	7 (9%)		21 (18%)	9 (8%)	
T2	107 (71%)	49 (62%)		79 (69%)	77 (67%)	
T3	20 (13%)	23 (29%)	***0.027***	14 (13%)	29 (25%)	***0.007***
**LNM**						
*N* (−)	75 (50%)	27 (34%)		62 (54%)	40 (35%)	
*N* (**+**)	75 (50%)	53 (67%)	***0.01***	52 (46%)	75 (65%)	***0.003***
**Tumor differentiation**						
Well	26 (17%)	11 (14%)		26 (23%)	11 (10%)	
Moderate	93 (62%)	41 (52%)		71 (62%)	63 (55%)	
Poor	31 (21%)	27 (34%)	0.082	17 (15%)	41 (35%)	***<0.001***
**ER**						
Negative	81 (54%)	46 (58%)		49 (43%)	78 (68%)	
Positive	69 (46%)	33 (42%)	0.541	65 (57%)	37 (32%)	***<0.001***
**PR**						
Negative	77 (51%)	50 (63%)		49 (43%)	78 (68%)	
Positive	73 (49%)	29 (37%)	0.084	65 (57%)	37 (32%)	***<0.001***
**TNM stage**						
I	29 (7%)	3 (4%)		21 (18%)	9 (8%)	
II	103 (69%)	44 (56%)		79 (69%)	77 (67%)	
III	36 (24%)	32 (41%)	***0.028***	14 (12%)	29 (25%)	***0.007***
**HER2 status**						
Negative	117 (78%)	54 (68%)		91 (80%)	80 (70%)	
Positive	33 (22%)	25 (32%)	0.111	23 (20%)	35 (30%)	0.074
**Neoadjuvant therapy**						
CMT	62 (41%)	39 (49%)		50 (44%)	51 (44%)	
No treatment	88 (59%)	40 (51%)	0.244	64 (56%)	64 (56%)	0.941
**Postoperative treatment**						
CMT	95 (63%)	46 (58%)		19 (17%)	68 (59%)	
CMT+R	28 (19%)	21 (27%)		73 (64%)	30 (26%)	
No treatment	27 (18%)	12 (15%)	0.373	22 (19%)	17 (15%)	***<0.001***

LNM, lymph node metastasis; ER, estrogen receptor; PR, progesterone receptor; TNM, tumor node metastasis; HER2, human epidermal growth factor receptor 2; CMT, chemotherapy; CMT+R, chemotherapy + radiotherapy. Boldface indicates P < 0.05.

Follow-up data were available for all 229 patients. After a mean and median follow-up of 27 and 60 months, respectively, disease progression was observed in 37.2% of patients. Survival analysis was performed to compare DFS between patients with low-grade TB and those with high-grade TB. The 5-year DFS rate for patients with low-grade or high-grade TB was 72.7% and 40.0%, respectively. Thus, high-grade TB was associated with worse DFS of cancer patients (**Figure 2G**). In molecular subgroup analyses, high-grade TB was related to poor outcomes in patients with HER2-positive tumors (*p* < 0.001) (**Figure 2H**), luminal A tumors (*p* = 0.038) (**Figure 2I**), and triple-negative BC (TNBC) tumors (*p* = 0.028) (**Figure 2J**), but not in luminal B subtypes (*p* = 0.237) (**Supplementary Figure S1**). After adjusting confounding factors, multivariate analysis revealed that T stage (T2: hazard ratio [HR] = 3.256, 95% CI = 1.013–10.462; T3: HR = 4.016, 95% CI = 1.195–13.492), LNM status (HR = 3.276, 95% CI = 1.857–5.778), tumor differentiation (poor: HR = 8.402, 95% CI = 2.403–26.926), HER2 (HR = 1.725; 95% CI = 1.083–2.748), and TB (HR = 1.871, 95% CI = 1.197–2.924) were independent prognostic factors of BC patients (**Table 3**).

**Table 3:** Multivariable analysis for 5-DFS.

Parameters	HR	95% CI	*p*-value	Parameters	HR	95% CI	*p*-value
**T stage**				**T stage**			
T1	1.000			T1	1.000		
T2	3.256	1.013–10.462	**0.048**	T2	3.170	0.987–10.186	0.053
T3	4.016	1.195–13.492	**0.025**	T3	3.866	1.153–12.969	0.029
**Tumor differentiation**				**Tumor differentiation**			
Well	1			Well	1		
Moderate	2.252	0.684–7.411	0.182	Moderate	2.18	0.665–7.151	0.199
Poor	8.042	2.403–26.926	**0.001**	Poor	7.23	2.169–24.102	***0.001***
**LNM**				**LNM**			
Negative	1.000			Negative	1.000		
Positive	3.276	1.857–5.778	**0.001**	Positive	3.122	1.776-5.488	0.001
**HER2**				**HER2**			
Negative	1.000			Negative	1.000		
Positive	1.725	1.083–2.748	**0.022**	Positive	1.725	1.083-2.748	***0.028***
**TB**				**TB-CSC type**			
Low grade	1.000			Type I. v*s* type II and III	0.316	0.164-0.608	***0.001***
High grade	1.871	1.197–2.924	**0.006**	Type IV vs. type II and III	1.776	1.085-2.907	***0.022***

LNM, Lymph node metastasis; HER2, human epidermal growth factor receptor-2; TB, tumor budding; CSCs, cancer stem cells. Boldface indicates P < 0.05.

### Subgroup Analysis of the Association of TB With DFS in BC Patients

The prognostic significance of TB for 5-year DFS was analyzed in each subgroup (**Figure 2K**). High-grade TB predicted a worse DFS of BC patients (HR = 2.69, 95% CI = 1.76–4.11). High-grade TB also significantly predicted a worse DFS in subgroups based on age (≤50 years) (HR = 3.09, 95% CI = 1.80–5.32), age (>50 years) (HR = 2.29, 95% CI = 1.14–4.61), postmenopausal status (HR = 1.86, 95% CI = 1.01–3.42), T2 (HR = 2.18, 95% CI = 1.29–3.70), T3 (HR = 2.95, 95% CI = 1.28–6.79), LNM positivity (HR = 2.37, 95% CI = 1.48–3.81), poor differentiation (HR = 2.08, 95% CI = 1.14–3.79), PR-positive group (HR = 3.04, 95% CI = 1.47–6.32), stage II (HR = 2.88, 95% CI = 1.55–5.36), HER2 negativity (HR = 2.18, 95% CI = 1.29–3.68), HER2 positivity (HR = 3.60, 95% CI = 1.69–7.66), neoadjuvant chemotherapy (CMT) (HR = 2.4, 95% CI = 1.37–4.21), no neoadjuvant therapy (HR = 2.78, 95% CI = 1.44–5.35), postoperative CMT (HR = 2.6, 95% CI = 1.44–4.71), and no postoperative treatment (HR = 4.73, 95% CI = 1.53–14.6) subgroup. However, no significant association was found in the other subgroups (**Figure 2K**).

### High-Grade TB Was Correlated With EMT and Stemness of Cancer

**Figure 3:**
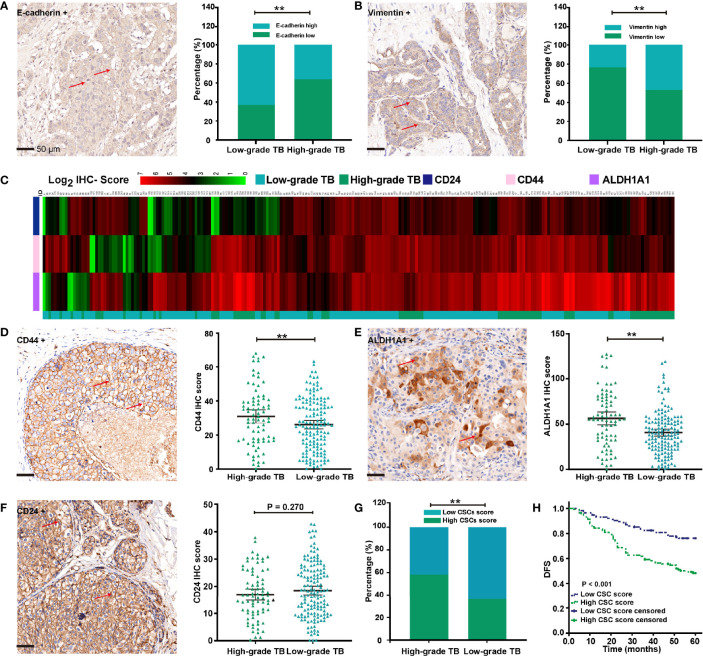
Associations of TB with EMT and tumor stemness. **(A)** Representative IHC images of E-cadherin and its associations with TB grade. **(B)** Representative IHC images of vimentin and its associations with TB grade. **(C)** Heatmap showed CSC marker expression in 229 cases of BC patients. **(D)** Representative IHC images of CD44 and its associations with TB grade. **(E)** Representative IHC images of ALDH1A1 and its associations with TB grade. **(F)** Representative IHC images of CD24 and its associations with TB grade. **(G)** Association between TB grade and CSC score. **(H)** The Kaplan-Meier survival curve shows disease-free survival of BC after stratification by CSC score. ^**^ *p* < 0.01.

IHC analysis of 229 cases of BC revealed that high-grade TB was significantly associated with low expression of E-cadherin (**Figure 3A**). As expected, vimentin was more likely to be upregulated in high-grade TB tissues (**Figure 3B**). These results demonstrate that TB is associated with the EMT process in BC patients.

Through the process of EMT, some detached cancer cells can adapt to a hypoxic environment and acquire resistance to anoikis to realize survival and metastasis (11, 12). Based on previous research, TB cells may acquire stem cell phenotypes to allow the colonization (3). Thus, expression of classic CSC markers, CD44, CD24, and ALDH1A1 was detected by IHC in TAMs (**Figure 3C**). As shown in **Figures 3D–F**, CD44 and CD24 were mainly located in the cell membrane, while ALDH1A1 was mainly located in the cytoplasm. We also found that CD44 and ALDH1A1 were more likely upregulated in high-grade TB tissues (**Figures 3D, E** **)**. No significant association was found between TB and CD24 expression (**Figure 3F**). We performed Cox regression analysis to establish a CSC score, consisting of three parameters (CD44, ALDH1A1, and CD24). The Cox regression coefficient of CD44, ALDH1A1, and CD24 are 0.029, 0.013, and −0.025, respectively. A formula, which is based on Cox regression coefficient of three CSC markers and IHC score of three CSC markers, was established to calculate CSC score. The CSC score is = 0.029 × (CD44 IHC score) + 0.013 × (ALDH1A1 IHC score) − 0.025 × (CD24 IHC score). According to the median value (0.89) of the CSC score, we classified BC patients into high and low CSC score groups. Among 229 patients, the CSC score was high in 115 patients (49%), and 114 patients (51%) were defined as low CSC score. Further research revealed that CSC score was significantly associated with T stage, LNM, tumor differentiation, ER positivity, PR positivity, and TB of BC (**Table 2** and **Figure 3G**). In contrast, no significant association was found between CSC score and other clinicopathological factors. Kaplan-Meier survival analysis showed that the duration of DFS of BC patients with low CSC scores was significantly longer than that of those with high CSC scores (*p* < 0.001) (**Figure 3H**).

### Combination of TB Grade and CSC Score Improves Prognostic Evaluation

Our data show that TB is an independent prognostic factor for BC, and that this complex biological behavior is closely related to CSC characteristics. Here, we also assessed the predictive value of the combination of TB grade and CSC score for 5-year DFS in BC patients.

**Figure 4:**
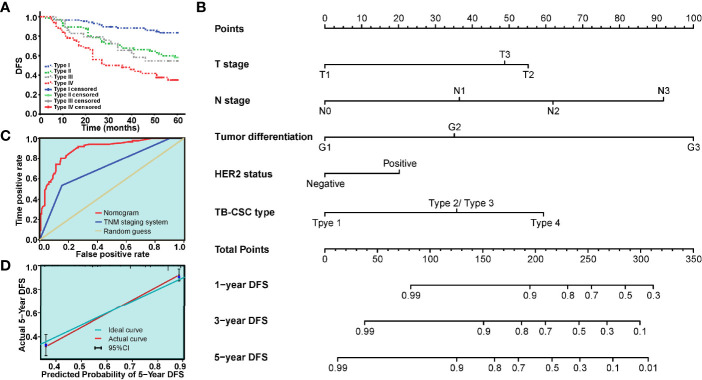
The model based on TB-CSC type for predicting tumor recurrence in patients with BC. **(A)** The Kaplan-Meier survival curve shows DFS after stratification by TB-CSC type. **(B)** The nomogram based on TB-CSC type predicting DFS probability of BC patients. **(C)** Calibration plot showing favorable agreement between the predicted rate (red line) and actual rate (green line). **(D)** The ROC curve shows a better prognostic value of nomogram on recurrence than TNM staging system.

Based on TB and CSC score, we classified patients into TB_low_-CSCs_low_ (type I), TB_low_-CSCs_high_ (type II), TB_high_-CSCs_low_ (type III), and TB_high_-CSCs_high_ (type IV) subgroups. Survival analysis revealed that patients in the type I group had the best DFS, while the worst DFS was found in the type IV group (**Figure 4A**). As the type II and type III groups had similar survival, we grouped these two types together for multivariable analysis. Multivariable Cox regression analysis of the relevant clinical variables and TB-CSC type revealed that TB-CSC type was an independent prognostic factor (**Table 3**).

A nomogram, integrating the TB-CSC type and clinicopathological risk variables was established to predict the probability of 1-year, 3-year, and 5-year DFS in BC patients (**Figure 4B**). The predictive accuracy of the nomogram for DFS is displayed in **Figure 4C**. The detailed points of each variable were provided in the following, T stage (T1: 0.0, T2: 55.2, T3: 48.8), N status (N0: 0.0, N1: 36.5, N2:61.9, N3: 91.9), histological grade (G1: 0.0, G2: 35.2, G3: 100.0), HER-2 status (negative: 0.0, positive: 20.2), and TB-CSC type (type 1: 0.0, type 2 or type 3: 35.8, type 4: 59.3). The c-index of this nomogram for 5-year DFS was 0.837 (95% CI = 0.76–0.92). Calibration curves showed that the models performed well compared with ideal models’ performance in both cohorts (**Figure 4C**). The nomograms also had better predictive ability than the TNM staging system, with area under the curve values of 0.892 (95% CI = 0.850–0.935) and 0.713 (95% CI = 0.644-0.783) (**Figure 4D**).

## Discussion

BC is a highly heterogeneous disease, with wide variation in prognosis among different molecular subtypes (31). Disease risk assessment to guide individualized treatment of cancer patients is particularly essential and urgent for precision medicine (32). As two different aspects of the tumor microenvironment, TB and CSCs are promising prognostic indicators for risk assessment. For its simple evaluation method and enormous clinical significance, TB is an emerging prognostic biomarker in solid cancers (33, 34). Likewise, the independent predictive significance of CSC markers in the prognosis of cancer has been documented (35–37). The current study revealed that high-grade TB was correlated with the TNM stage, LNM, EMT, and CSC score of BC patients. Furthermore, we demonstrated that TB was an independent prognostic factor, and that high-grade TB was correlated with worse DFS of cancer patients. Finally, a novel nomogram based on TB grade and CSC score was constructed and shown to improve the prognostic evaluation of BC patients.

Accurate assessment of the TB is the key to fully exploiting its prognostic value. Hematoxylin and eosin staining of specimens is typically used to assess TB; however, it is challenging to accurately identify TB by this method against a background of peritumoral inflammation. Pan-cytokeratin IHC, a powerful approach that can highlight tumor buds and reduce observed differences, has been adopted to assess TB (38). In our study, TB was verified to be an independent prognostic factor in BC. We also demonstrated that TB was associated with age, menopausal status, T stage, TNM stage, and LNM status. Consistent with previous studies (39), our study showed that TB was an independent prognostic factor of BC patients. In BC, which is highly heterogeneous, prognosis varies widely among different subtypes. Our research verified that high-grade TB predicted a worse DFS in patients with HER2+ tumors, luminal A tumors, and TNBC tumors. However, no significant association was found between TB and luminal B subtypes. Subgroup analysis also demonstrated that the prognostic value of TB varies widely among different subgroups. Thus, the prognostic value of TB may be different in different subtypes and subgroups.

The EMT process, which provides tumor cells with several prometastatic traits (40, 41), has also been implicated in the metastatic process (42). Generally, epithelial-type cells can gain more mesenchymal traits to increase their invasive ability *via* EMT (43), thereby overcoming antimetastatic bottlenecks and achieving the great potential for metastasis. In our research, diminished expression of E-cadherin was found in the high-grade TB sample and aberrantly expressed vimentin was observed in the low-grade TB samples. High-grade TB was also associated with EMT of BC patients. TB correlates with EMT confirmed the hypothesis that TB may represent the EMT process. Through EMT, some detached cancer cells from the primary site could acquire stem cell phenotype to adapt to a hypoxic environment (26, 27). Thus, TB cells might acquire stem cell phenotypes to realize distant metastasis and colonization (28). As expected, we demonstrated that high-grade TB was highly correlated with overexpression of CSC markers in BC. Furthermore, we found that CD44 and ALDH1A1 were strongly expressed in tumor buds. No significant association was found between TB grade and CD24 expression.

Our study of TB and CSC markers inspires us a new understanding of molecular and pathogenetic mechanisms of TB, which could be a potential target of “antibudding therapies”. As part of the invasive tumor front, TB should be integrated into the biological context for better characterized. The role of CSC score as a prognostic factor is emerging. In esophageal cancer (44), high CSC score predicted a worse overall survival of cancer patients. In the current research, the CSC score integrated three types of CSC markers (CD24+, CD44+, ALDH1A1+). For the first time, we found that a high CSC score predicted worse DFS of BC patients. A retrospective analysis found that a CSC-related signature could facilitate the prognostic prediction in pancreatic ductal adenocarcinoma (45), consistent with our results. However, almost no study has explored the interaction between TB and CSC score. For the first time, we verified that high-grade TB was correlated with high CSC score. We also revealed that CSC score was significantly associated with tumor sizes, LNM, tumor differentiation, ER, PR, and TB. Consequently, tumor classification based on TB and CSC score revealed that TB_low_-CSCs_low_ (type I) patients had the best 5-year DFS, whereas TB_high_-CSCs_high_ group had the worst 5-year DFS. For the first time, we combined TB and CSC score to evaluate prognosis. This method paves a new way to potential new tumor therapies.

Due to the limitations of a single prognostic factor, an integrated prognostic system was needed for a better prognostic evaluation. In previous research, autophagy-, EMT-, and immune-related gene signatures of cancers have been extensively reported (46–48), while few studies have combined CSC expression profile and TB to conduct risk assessment. The nomogram is a comprehensive predictive model, which assigns a score to each risk factor based on its contribution to the prognosis. The incidence rate was then evaluated through the scoring system. Here, we developed a novel predictive nomogram (49) for recurrence in invasive BC; the first TB-CSC-based nomogram in BC was established. The result demonstrated that TB-CSC-based nomograms could provide a more accurate prognostic assessment than the TNM staging system.

However, this study had some limitations. Firstly, as it was a retrospective study with a relatively small sample size, it was difficult to exclude heterogeneity and define optimal cutoff value. In the same cancer type, cutoff value of TB often varies widely in different researchers (50, 51). Thus, further validation is needed in large-scale multicenter randomized controlled trials. We also hope that further results about TB will be uploaded to a public database (such as The Cancer Genome Atlas), which could provide doctors with global dataset and optimal cutoff value of TB and CSCs to evaluate prognosis. Second, although pan-cytokeratin IHC exhibited its excellent score ability, more accurate and convenient methods are needed to be combined to assess TB, such as artificial intelligence tools (52). Third, although tissue cores from different areas were used to construct the TMAs, not every core of the TMAs could completely represent the optimal site for TB assessment. In this sense, slides of the whole tumor will be of great importance to assess TB. Fourth, the size of the TB needs to be strictly uniformly characterized in future research. In some studies, TB was defined as a cell cluster of less than four cells, whereas other studies used a threshold of five or more cells.

Despite the aforementioned limitations, this study found high-grade TB was correlated with TNM stage, LNM, and EMT of BC. Furthermore, we found that TB was an independent prognostic factor, and that high-grade TB correlated with worse DFS of cancer patients. We then revealed that CSC score (based on CD44, CD24, and ALDH1A1) was correlated with tumor progression and TB. A novel nomogram based on TB and CSC score, which improved the prognostic evaluation of BC, was constructed. The defined subtype may provide doctors a candidate guideline for individualized treatment of cancer patients.

## Data Availability Statement

The original contributions presented in the study are included in the article/**Supplementary Material**. Further inquiries can be directed to the corresponding authors.

## Ethics Statement

Written informed consent was obtained from the individual(s) for the publication of any potentially identifiable images or data included in this article.

## Author Contributions

Participated in research design: ZX, QH, QX, and BX. Performed data analysis: ZX, QH, and QX. Experimental operation: ZX, QH, and QX. Wrote or contributed to the writing of the manuscript: QH, ZX, and BX. All authors contributed to the article and approved the submitted version.

## Funding

This work was supported by the National Natural Science Foundation of China (No. 82103005, No. 81872376).

## Conflict of Interest

The authors declare that the research was conducted in the absence of any commercial or financial relationships that could be construed as a potential conflict of interest.

## Publisher’s Note

All claims expressed in this article are solely those of the authors and do not necessarily represent those of their affiliated organizations, or those of the publisher, the editors and the reviewers. Any product that may be evaluated in this article, or claim that may be made by its manufacturer, is not guaranteed or endorsed by the publisher.

## Abbreviations

BC, breast cancer; TB, tumor budding; LNM, lymph node metastasis; TNM, tumor node metastasis; EMT, epithelial-mesenchymal transition; DFS, disease-free survival; CSCs, cancer stem cells; ER, estrogen receptor; PR, progesterone receptor; HER2, human epidermal growth factor receptor 2; AJCC, American Joint Committee on Cancer; TNBC, triple-negative BC; CMT, chemotherapy; hormone receptor positive, estrogen receptor positive or progesterone receptor positive; hormone receptor negative, estrogen receptor negative and progesterone receptor negative; TMAs, tissue microarrays; CMT+R, chemotherapy + radiotherapy.
