# Supplementary figures and images for: Breast Cancer Classification Based on Tumor Budding and Stem Cell-Related Signatures Facilitate Prognosis Evaluation
Source: Front Oncol. 2022 Jan 10;11:818869. doi: 10.3389/fonc.2021.818869 (PMC8784696; doi:10.3389/fonc.2021.818869)

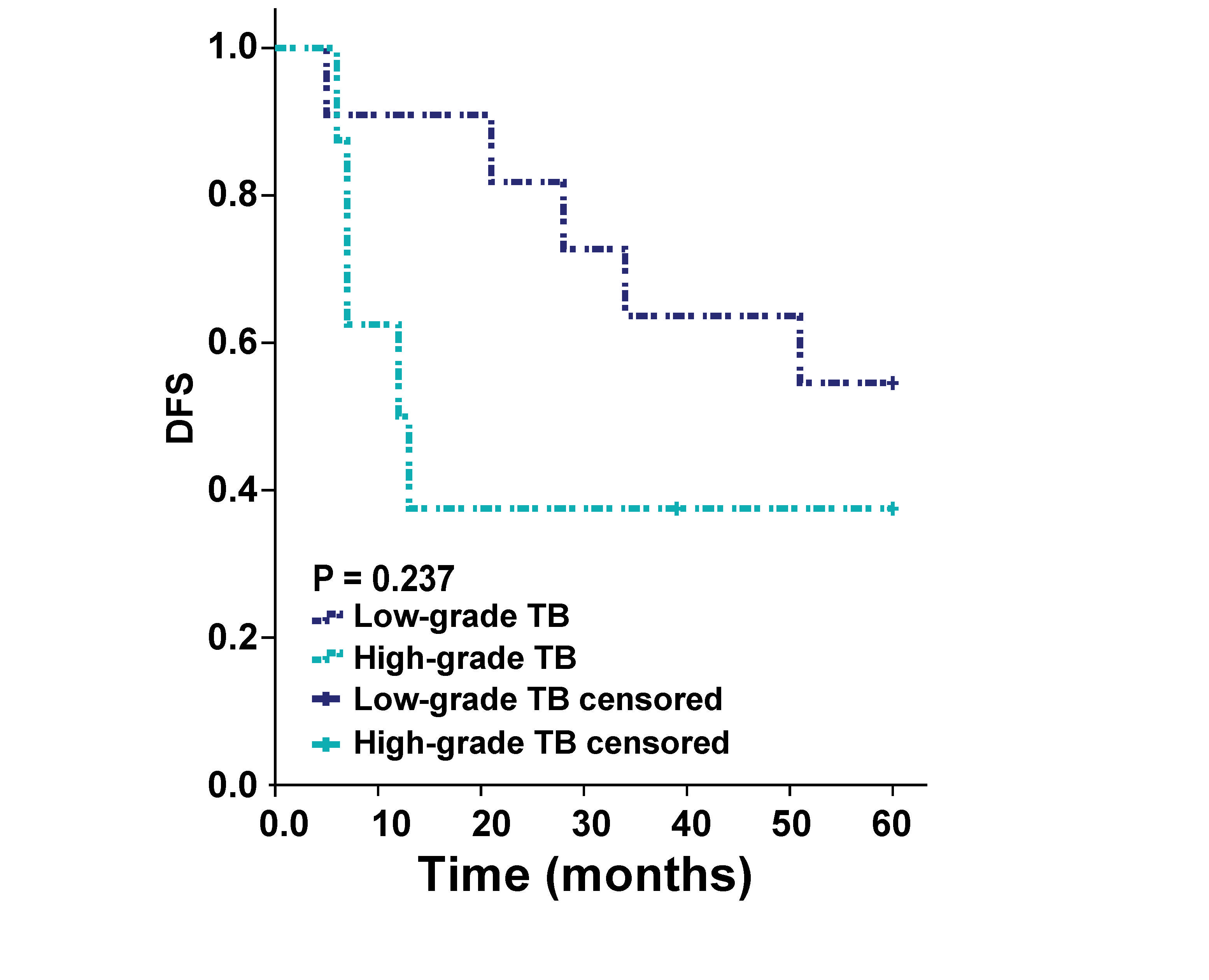

Supplement: Supplementary file 1 [file Image_1.tif]
